# The Aβ1–42/Aβ1–40 ratio in CSF is more strongly associated to tau markers and clinical progression than Aβ1–42 alone

**DOI:** 10.1186/s13195-022-00967-z

**Published:** 2022-02-01

**Authors:** Constance Delaby, Teresa Estellés, Nuole Zhu, Javier Arranz, Isabel Barroeta, María Carmona-Iragui, Ignacio Illán-Gala, Miguel Ángel Santos-Santos, Miren Altuna, Isabel Sala, M. Belén Sánchez-Saudinós, Laura Videla, Sílvia Valldeneu, Andrea Subirana, Mireia Tondo, Francisco Blanco-Vaca, Sylvain Lehmann, Olivia Belbin, Rafael Blesa, Juan Fortea, Alberto Lleó, Daniel Alcolea

**Affiliations:** 1 Sant Pau Memory Unit, Department of Neurology, Institut d’Investigacions Biomèdiques Sant Pau - Hospital de Sant Pau, Universitat Autònoma de Barcelona, Sant Antoni Maria Claret 167, 08025 Barcelona, Spain; 2 IRMB, INM, Université de Montpellier, INSERM, CHU de Montpellier, Laboratoire de Biochimie-Protéomique clinique, Montpellier, France; 3 Centro de Investigación Biomédica en Red en Enfermedades Neurodegenerativas, CIBERNED, Madrid, Spain; 4 Fundació Catalana Síndrome de Down, Centre Mèdic Down, Barcelona, Spain; 5 Servei de Bioquímica, Institut d’Investigacions Biomèdiques Sant Pau - Hospital de Sant Pau, Universitat Autònoma de Barcelona, Barcelona, Spain; 6 Servei de Bioquímica i Biologia Molecular, Unversitat Autònoma de Barcelona, Barcelona, Spain; 7 Centro de Investigación Biomédica en Red en Diabetes y Enfermedades Metabólicas (CIBERDEM), Madrid, Spain

**Keywords:** Amyloid, Aβ1–40, Aβ1–42, Cerebrospinal fluid, Tau, Biomarkers

## Abstract

**Background:**

Cerebrospinal fluid (CSF) Aβ1–42 levels and the Aβ1–42/Aβ1–40 ratio are markers of amyloid pathology, but previous studies suggest that their levels might be influenced by additional pathophysiological processes.

**Aims:**

To compare Aβ1–42 and the Aβ1–42/Aβ1–40 ratio in CSF in different neurodegenerative disorders and study their association with other biomarkers (tTau, pTau181, and NfL) and with cognitive and functional progression.

**Methods:**

We included all participants from the Sant Pau Initiative on Neurodegeneration (SPIN) with CSF Aβ1–42 and Aβ1–42/Aβ1–40. Participants had diagnoses of Alzheimer’s disease (AD), dementia with Lewy bodies, frontotemporal lobar degeneration-related syndromes, non-neurodegenerative conditions, or were cognitively normal. We classified participants as “positive” or “negative” according to each marker. We compared CSF levels of tTau, pTau181, and NfL between concordant and discordant groups through ANCOVA and assessed differences in cognitive (MMSE, FCSRT) and functional (GDS, CDR-SOB) progression using Cox regression and linear-mixed models.

**Results:**

In the 1791 participants, the agreement between Aβ1–42 and Aβ1–42/Aβ1–40 was 78.3%. The Aβ1–42/Aβ1–40 ratio showed a stronger correlation with tTau and pTau181 than Aβ1–42 and an agreement with tTau and pTau181 of 73.1% and 77.1%, respectively. Participants with a low Aβ1–42/Aβ1–40 ratio showed higher tTau and pTau181 and worse cognitive and functional prognosis, regardless of whether they were positive or negative for Aβ1–42. The results were consistent across stages, diagnostic categories, and use of different cutoffs.

**Conclusion:**

Although Aβ1–42 and Aβ1–42/Aβ1–40 are considered markers of the same pathophysiological pathway, our findings provide evidence favoring the use of the Aβ1–42/Aβ1–40 ratio in clinical laboratories in the context of AD.

**Supplementary Information:**

The online version contains supplementary material available at 10.1186/s13195-022-00967-z.

## Introduction

Cerebrospinal fluid (CSF) biomarkers of Alzheimer’s disease (AD) have changed the management of patients with cognitive impairment [1]. In particular, CSF levels of β-amyloid 1–42 (Aβ1–42), total tau (tTau), and its phosphorylated form on threonine 181 (pTau181) have shown very high accuracy for the diagnosis of AD [2–4]. They are consequently being implemented in clinical laboratories [5–9] both for diagnosis and research settings, as well as in clinical trials.

The role of Aβ1–42 in CSF as a marker of amyloid pathology is widely accepted [6, 9, 10]. However, a series of studies have shown that reduced levels of CSF Aβ1-42 can also be found in a variety of conditions different from AD, such as inflammatory diseases, prionopathies, amyloid angiopathy, or frontotemporal dementia [10–15]. The Aβ1–42/Aβ1–40 ratio has proven to be of great value in detecting amyloid pathology both in CSF [16, 17] and plasma [18, 19] and has shown a better correlation with amyloid burden in PET than Aβ1–42 alone [20–22]. However, Aβ1–40 levels are not systematically assessed in many clinical laboratories alleging that this marker alone has no diagnostic value. A large-scale head-to-head comparison between Aβ1–42 and the Aβ1–42/Aβ1–40 ratio would address the question of whether these two markers are equally tracking the same pathophysiological process. It would also inform laboratories on whether to implement the Aβ1–42/Aβ1–40 ratio into the clinical routine.

In the present work, we studied the CSF markers Aβ1–42 and Aβ1–42/Aβ1–40 ratio in a large cohort of participants with a variety of neurodegenerative disorders. We compared the agreement between both measures in different contexts and studied their association with other CSF biomarkers (tTau, pTau181, and NfL) and with cognitive and functional progression. This information is highly relevant in the implementation and interpretation of these markers in clinical routine.

## Material and methods

### Study participants and clinical classification

We included all participants in the Sant Pau Initiative on Neurodegeneration (SPIN) cohort [23] that underwent lumbar puncture for CSF biomarkers between November 2013 and August 2021. The diagnostic groups included patients with mild cognitive impairment (MCI) or dementia and with either pathophysiological evidence of Alzheimer’s disease (AD), frontotemporal lobar degeneration (FTLD)-related syndromes [24, 25], or probable dementia with Lewy bodies (DLB). Diagnoses were established following internationally accepted diagnostic criteria [6, 7, 9, 26–28]. Clinical symptoms, neuroimaging and CSF biomarkers were considered for the diagnostic classification of patients. We also included participants with Down syndrome (DS) [29, 30] and cognitively normal controls (CN). All CN participants had normal cognitive scores in a formal neuropsychological evaluation [23, 31]. Patients with other diagnoses were grouped as “others” and included participants with prionopathy and other non-neurodegenerative conditions such as psychiatric etiology, vascular cognitive impairment, inflammatory, and those with uncertain etiology. Details about the SPIN cohort have been reported previously [23].

### CSF collection and analysis

CSF was obtained by lumbar puncture, collected, and processed in polypropylene tubes following international recommendations [32, 33]. The same pre-analytical handling was followed in all samples [23]. Concentrations of Aβ1–42, Aβ1–40, total tau (tTau), and 181-phosphorylated tau (pTau181) in CSF were measured using commercially available kits in the Lumipulse fully automated platform (Fujirebio-Europe), as previously described [22, 34], and following provider’s instructions in line with Global Biomarker Standardization [35, 36]. For each sample, Aβ1–42, Aβ1–40, tTau, and pTau181 were quantified simultaneously in the same run immediately after the first freeze-thaw cycle of each sample using pristine aliquots containing 500 μL of CSF [22, 34]. The results of the Lumipulse G β-amyloid 1–42 have been standardized according to certified reference material developed by the International Federation of Clinical Chemistry and Laboratory Medicine as recommended by their working group for CSF proteins. Our laboratory participates in the Alzheimer’s Association Quality Control Program led by the University of Gothenburg [37]. Three levels of internal quality controls provided by the manufacturer were assessed for each analyte to assess the reproducibility of the assays. We included at least one level of quality control per analyte in each run. Inter-assay coefficients of variation (CV%) were between 1.7% and 6.8% for all levels and analytes.

Neurofilament light (NfL) levels in CSF were measured using a commercially available ELISA kit (NF-light, UMAN DIAGNOSTICS, Umea, Sweden) as previously described [24, 25]. The mean inter- and intra-assay coefficients of variation were 3.4% and 11.4%, respectively.

### Definition of amyloid profile

To ensure that the cutoffs for Aβ1–42 and Aβ1–42/Aβ1–40 had comparable levels of sensitivity and specificity, we applied cutoffs corresponding to one-sided 95% quantile (Q95%) values in a middle-aged cognitively healthy population (age range 23–60 years, 67% female). This age range was selected to minimize the presence of preclinical AD in the reference population. We used these Q95% cutoffs for Aβ1–42 (637 pg/mL) and Aβ1–42/Aβ1–40 (0.070) to classify all participants in four different profiles: two concordant profiles in which both Aβ1–42 and Aβ1–42/Aβ1–40 ratio were above (Aβ1–42[−]Ratio[−]) or below (Aβ1–42[+]Ratio[+]) their respective cutoffs, and two discordant profiles, in which only one of the two amyloid parameters, Aβ1–42 or the Aβ1–42/Aβ1–40 ratio, was abnormal (Aβ1–42[+]Ratio[−] and Aβ1–42[−]Ratio[+], respectively). The objective of classifying participants in four amyloid profiles is to assess the particularities of those groups where Aβ1–42 and the Aβ1–42/Aβ1–40 ratio are discordant (Aβ1–42[−]Ratio[+] and Aβ1–42[+]Ratio[−]) and compare them to those that are clearly amyloid negative (Aβ1–42[–]Ratio[−]) or clearly amyloid positive (Aβ1–42[+]Ratio[+]) according to both markers. More details about the cognitively healthy reference population and results after applying other cutoffs can be found in Additional file 1.

### Measures of cognitive and functional impairment

Cognition was assessed by the Mini-Mental State Examination (MMSE) and the free and cued selective reminding test (FCSRT). Global functional impairment was assessed by the Clinical Dementia Rating Scale Sum of Boxes (CDR-SOB) and the Global Deterioration Scale (GDS). Outcomes for cognitive and functional impairment were defined as MMSE < 24 and GDS ≥ 4, respectively [31].

### APOE genotyping

DNA was extracted from whole blood using standard procedures and *APOE* was genotyped according to previously described methods [38].

### Statistical analysis

Non-normally distributed variables were log-transformed. Differences in the frequency of categorical variables were assessed by the *χ*^2^ test, and we used age- and sex-adjusted analysis of covariance (ANCOVA) to compare CSF levels of tTau, pTau181, and NfL between concordant and discordant groups. We determined Spearman’s correlation coefficients between biomarkers in the whole sample and after stratification by diagnostic category, clinical stage, and amyloid profile. We assessed the association with cognitive and functional progression in patients with mild cognitive impairment through Kaplan-Meier survival curves and age- and sex-adjusted Cox regression analysis. We studied the association of amyloid profiles with a cognitive decline through linear-mixed models. The initial model included baseline MMSE score, baseline age, sex, years of education, pTau181 levels, diagnosis, time, *APOE4* status, and amyloid profile together with its interaction with time and with *APOE4* status as fixed factors. We defined random intercepts for diagnosis and at the participant level to account for repeated measures and modeled residual errors per diagnostic group. The alpha threshold was set at 0.05, and all analysis were performed using MEDCALC (MEDCALC software ver 15.2.2) and packages “survival” (v.3.1-12), “survminer” (v.0.4.6), “nlme” (v.3.1-147), “multcomp” (v.1.4-13), “ggplot2” (v.3.3.0), and “ggpubr” (v.0.3.0), as implemented in the R statistical software (v 3.6.2). The alpha threshold was set at 0.05 for all analyses.

### Ethical approval and consent to participate

All procedures in the study were approved by the Sant Pau Ethics Committee following the standards for medical research in humans recommended by the Declaration of Helsinki. All participants or their legally authorized representatives gave written informed consent.

## Results

### Demographics and core CSF biomarkers

We included a total of 1791 participants from the SPIN cohort. The demographic characteristics and biomarker results are summarized in Table 1. There were differences in age and male/female proportion between the groups. As expected, the *APOEε4* allele was more frequent in AD patients (50%; *p* < 0.001), and no differences were observed among the other groups. MMSE scores were lower in all symptomatic groups compared to CN (*p* < 0.001).

**Table 1:** Demographics, clinical information, and biomarkers across diagnostic categories

		CN	AD	DLB	FTLD	Down	Others
***N***		197	518	128	186	225	536
**AGE, years**	Mean (SD)	53.5 (12.5)	73.1 (6.88)	75.7 (5.49)	70.8 (8.58)	45.1 (10)	70 (9.18)
	Median [IQR]	55 [46–62]	74 [69–78]	76 [71–80]	72 [66–77]	48 [40–52]	71 [65–77]
**SEX, females/males (% females)**		132/65 (67%)	311/207 (60%)	64/64 (50%)	77/109 (41.4%)	103/122 (45.8%)	310/226 (57.8%)
**MMSE score**	Mean (SD)	29.2 (0.889)	23.6 (4.52)	24 (4.09)	24 (5.08)	NA^a^	25.4 (4.12)
	Median [IQR]	29 [29–30]	25 [22–27]	24.5 [22–27]	25 [21–28]	NA^a^	27 [24–28]
**Education, years**	Mean (SD)	15.6 (3.99)	10.7 (4.72)	9.18 (5.05)	12 (5.13)	15.3 (3.07)	10.7 (4.93)
	Median [IQR]	16 [12–20]	10 [8–13]	8 [7–12]	12 [8–16]	NA^a^	9 [8–13]
**APOEε4, APOEε4−/APOEε4+ (%APOEε4+)**		46/151 (23.4%)	254/251 (50.3%)	33/93 (26.2%)	37/139 (21%)	44/177 (19.9%)	115/413 (21.8%)
**Follow-up, years**	Mean (SD)	2.02 (1.99)	1.09 (1.49)	3.33 (1.93)	1.71 (1.47)	1.76 (2.09)	0.59 (1.23)
	Median [IQR]	1.71 [0–2.82]	0 [0–2.06]	3.46 [2.07–4.52]	1.52 [0.242–2.66]	NA^a^	0 [0–0.383]
**Aβ1–42, pg/ml**	Mean (SD)	1148 (397)	562 (165)	817 (399)	938 (446)	715 (417)	1000 (500)
	Median [IQR]	1118 [849–1371]	556 [432–673]	703 [542–1009]	850 [569–1229]	583 [430–892]	896 [609–1323]
**Aβ1–40, pg/ml**	Mean (SD)	11,694 (3595)	12,790 (3781)	11,506 (4189)	10,806 (4357)	11,673 (4678)	11,399 (4390)
	Median [IQR]	11,329 [9238–13,777]	12,541 [10,125–15,122]	10,885 [8882–14,234]	10,140 [7710–13,334]	11,035 [8346–14,594]	10,638 [8112–13,986]
**Aβ1–42/Aβ1–40**	Mean (SD)	0.0991 (0.0181)	0.0453 (0.0108)	0.0727 (0.0263)	0.0877 (0.0221)	0.0615 (0.0226)	0.0862 (0.0225)
	Median [IQR]	0.104 [0.0986–0.109]	0.0445 [0.0376–0.0514]	0.0662 [0.0503–0.0996]	0.0964 [0.0731–0.103]	0.0562 [0.0422–0.078]	0.0943 [0.0665–0.103]
**tTau, pg/ml**	Mean (SD)	255 (152)	748 (358)	456 (334)	387 (260)	644 (520)	334 (231)
	Median [IQR]	230 [174–291]	656 [488–915]	361 [253–525]	322 [222–456]	489 [262–870]	292 [213–378]
**pTau181, pg/ml**	Mean (SD)	37.3 (27.3)	122 (60.3)	70.5 (55.5)	49.9 (39.2)	100 (96.8)	45 (27.4)
	Median [IQR]	31.6 [24.9–42]	105 [78.7–145]	51 [35.7–83]	39.7 [29.2–54.1]	63.6 [29.8–151]	41 [29.3–52.7]
**NfL, pg/ml**	Mean (SD)	475 (256)	1330 (1824)	1108 (570)	2079 (1836)	815 (773)	1488 (1340)
	Median [IQR]	458 [320–533]	981 [791–1254]	918 [719–1297]	1412 [884–2767]	614 [357–1014]	1089 [595–1878]
**Clinical stage, CN/MCI/dementia (% MCI)**		177/0/0	0/296/208	2/60/64	5/90/80	NA^a^	27/348/125
**Amyloid profile (Aβ1–42[−]Ratio[−]/Aβ1–42[−]Ratio[+]/Aβ1–42[+]Ratio[−]/Aβ1–42[+]Ratio[+])**		165/13/11/8	4/154/2/358	50/29/7/42	120/8/23/35	61/37/13/114	345/45/47/100

*MMSE* Mini-Mental State Examination, *CSF* cerebrospinal fluid, *CN* cognitively normal, *AD* Alzheimer’s disease, *DLB* dementia with Lewy bodies; *FTLD* frontotemporal lobar degeneration-related syndrome, *MCI* mild cognitive impairment

^a^Due to specific particularities of the clinical and cognitive assessment in the context of intellectual disability, participants with Down syndrome were excluded from the prognostic analysis

### Aβ1–42 and the Aβ1–42/Aβ1–40 ratio in CSF show high but not perfect agreement

**Fig. 1:**
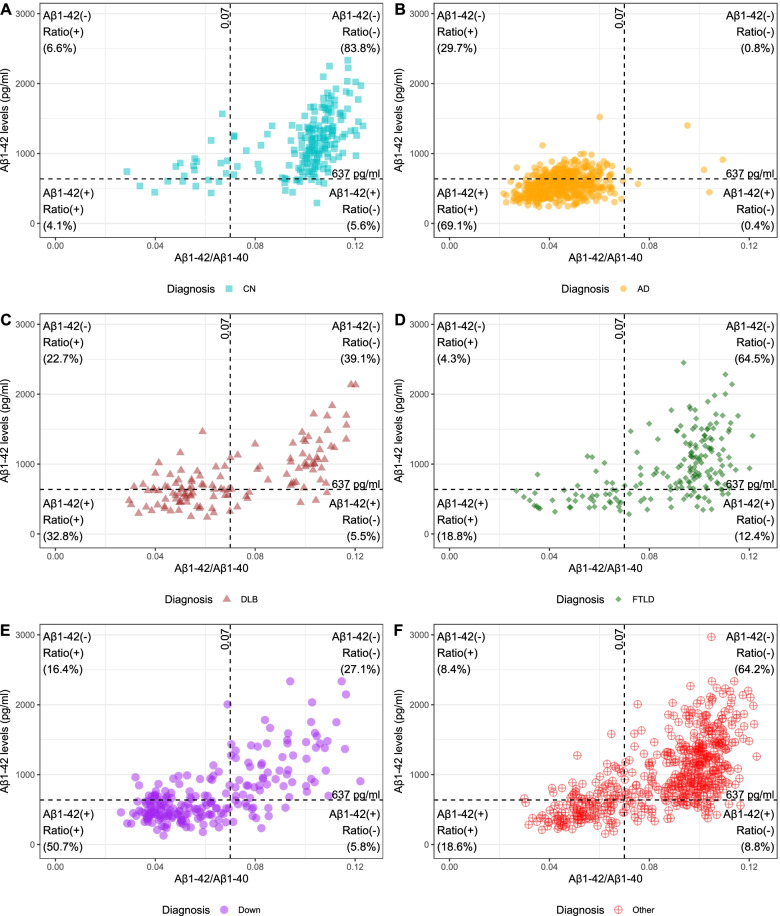
Distribution of participants according to CSF levels of Aβ1–42 and amyloid ratio within diagnostic categories. CN, cognitively normal; AD, Alzheimer’s disease; DLB, dementia with Lewy bodies; FTLD, frontotemporal lobar degeneration-related syndrome. Dashed lines indicate 95% quantile values (Q95%) for Aβ1–42 and Aβ1–42/Aβ1–40 in a middle-aged cognitively normal population as described in the “Material and methods” section

Figure 1 shows the distribution of participants based on their CSF Aβ1–42 and Aβ1–42/Aβ1–40 ratio measures. The correlation between these two parameters was rho = 0.71, *p* < 0.001. Using Q95% cutoffs as described in the “Material and methods” section, Aβ1–42 and Aβ1–42/Aβ1–40 ratio had an overall agreement of 78.3% in the whole sample, as both measures were normal (Aβ1–42[−]Ratio[−]) in 41.6% and both were abnormal (Aβ1–42[+]Ratio[+]) in 36.7% of participants. Within each diagnostic category, the agreement ranged from 69.9% (AD group) to 87.9% (CN group). More details about the agreement after applying other cutoffs values and with other biomarkers can be found in Additional file 1.

### Aβ1–42/Aβ1–40 ratio is more strongly associated with tau markers than Aβ1–42

We next studied the association of Aβ1–42 and the Aβ1–42/Aβ1–40 ratio with the other CSF biomarkers by assessing Spearman correlations in the whole sample and within diagnostic groups. In the whole sample, Aβ1–42 showed a significant correlation with tTau (rho = − 0.25, *p* < 0.001) and pTau181 (rho = − 0.32, *p* < 0.001). These correlations were stronger for the Aβ1–42/Aβ1–40 ratio (rho = − 0.69 and rho = − 0.75, respectively, both *p* < 0.001). Both Aβ1–42 and the Aβ1–42/Aβ1–40 ratio showed a similar correlation with NfL (rho = − 0.26 and rho = − 0.32, respectively, both *p* < 0.001). These stronger associations of Aβ1–42/Aβ1–40 ratio with tTau and pTau181 were observed within all symptomatic diagnostic categories (Additional file 1).

**Fig. 2:**
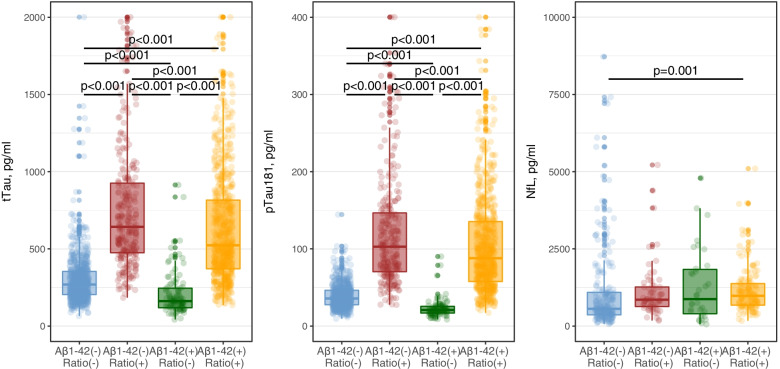
Levels of tTau (**A**), pTau181 (**B**), and NfL (**C**) in CSF according to their amyloid profile

Next, we compared CSF levels of tTau, pTau181 and NfL between all four amyloid profiles. The objective of this analysis was to assess the particularities of those groups where the Aβ1–42 and the Aβ1–42/Aβ1–40 ratio are discordant. As expected, compared to the Aβ1–42[−]Ratio[−] group and after adjusting by age and sex, the Aβ1–42[+]Ratio[+] profile was associated with higher levels of tTau (Tukey post hoc *p* < 0.001), pTau181 (Tukey post hoc *p* < 0.001), and NfL (Tukey post hoc *p* = 0.001). But we also found differences in tTau and pTau181 levels between the two discordant profiles. As seen in Fig. 2, Aβ1–42[−]Ratio[+] participants showed higher levels of tTau (Tukey post hoc *p* < 0.001) and pTau181 (Tukey post hoc *p* < 0.001) compared to those with Aβ1–42[+]Ratio[−]. These differences were also observed within all diagnostic categories and in all clinical stages (Additional file 1). These results indicate that reduced levels of the Aβ1–42/Aβ1–40 ratio are associated with high levels of CSF tau markers, regardless of the status of Aβ1–42 alone.

### Aβ1–42/Aβ1–40 ratio is more strongly associated with cognitive and functional progression than Aβ1–42

**Fig. 3:**
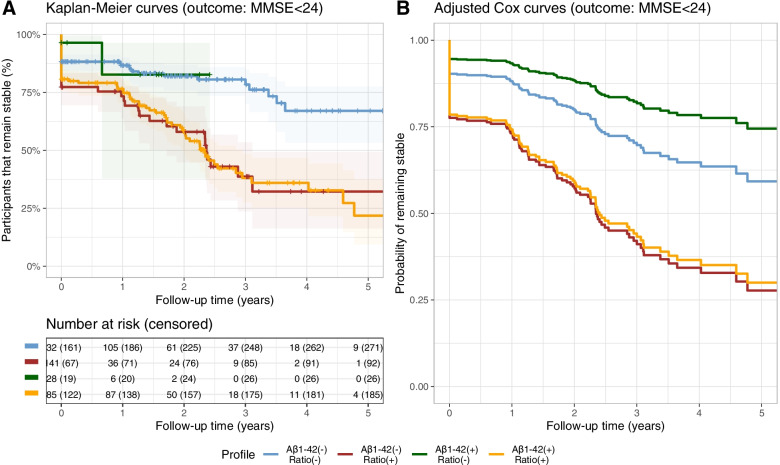
Cognitive progression in patients with mild cognitive impairment according to their amyloid profile. **A** Kaplan-Meier curve and **B** age- and sex-adjusted Cox regression display the risk of cognitive progression of all four amyloid profiles (outcome defined as MMSE < 24)

We studied the association of amyloid profiles with cognitive and functional progression in patients with MCI (*n* = 794) through Kaplan-Meier survival curves and age- and sex-adjusted Cox regression analysis. Supplementary Table 3 describes the characteristics of this subgroup. Due to specific particularities of the clinical and cognitive assessment in the context of intellectual disability, participants with Down syndrome were not included in this analysis. As displayed in Fig. 3, we found that patients with MCI with a low Aβ1–42/Aβ1–40 ratio had worse cognitive outcomes reflected by an earlier decline in MMSE scores. Compared to the Aβ1–42[−]Ratio[−] group, the adjusted risk of presenting a MMSE score lower than 24 during follow-up was 1.77 (1.25–2.49) times higher in the Aβ1–42[−]Ratio[+] group, 1.78 (1.33–2.39) times higher in the Aβ1–42[+]Ratio[+], but not different in the Aβ1–42[+]Ratio[−] group (*p* = 0.28). Similarly, the adjusted risk of progression to dementia was 1.55 (0.96–2.50) times higher in the Aβ1–42[−]Ratio[+] group and 2.07 (1.43–2.99) times higher in the Aβ1–42[+]Ratio[+] group compared to that of the Aβ1–42[−]Ratio[−]. The adjusted risk of progression to dementia in the Aβ1–42[+]Ratio[−] group was not significantly different from the Aβ1–42[−]Ratio[−] group (*p* = 0.26).

**Fig. 4:**
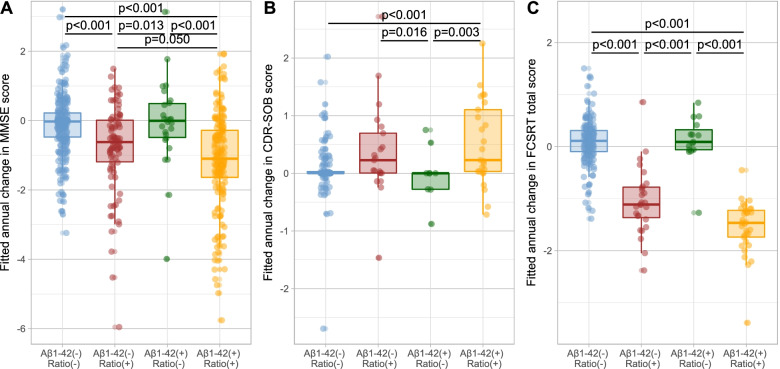
Estimation of the annual change in cognitive and functional scores across amyloid profiles. Estimations of the annual change in MMSE (**A**), CDR-SOB (**B**), and FCSRT total score (**C**) were calculated through linear-mixed models adjusted by baseline MMSE score, baseline age, sex, years of education, pTau181 levels, *APOEε4* status, and diagnosis. MMSE, Mini-Mental State Examination; CDR-SOB, Clinical Dementia Rating Sum of Boxes; FCSRT, free and cued selective reminding test

We also fitted linear-mixed models to assess the changes in longitudinal cognitive and functional measures by amyloid profiles. As shown in Fig. 4, after adjusting by baseline MMSE score, baseline age, sex, years of education, pTau181 levels, *APOEε4* status and diagnosis, participants with low Aβ1–42/Aβ1–40 ratio presented larger decreases in MMSE scores. The model estimated an annual decrease of − 1.32 (− 1.55 to − 1.09) points when Aβ1–42 was low and of − 0.86 (− 1.18 to − 0.55) points when Aβ1–42 was in the normal range. However, the annual change in MMSE scores in the two groups with normal Aβ1–42/Aβ1–40 ratio was not significantly different from zero. As displayed in Fig. 4, we also found that both groups with low Aβ1–42/Aβ1–40 had larger annual decreases in the FCSRT total score, estimated in − 1.21 in Aβ1–42[−]Ratio[+] and − 1.5 in Aβ1–42[+]Ratio[+], compared to 0.05 in Aβ1–42[−]Ratio[−] and 0.1 in Aβ1–42[+]Ratio[−]. Positivity in the amyloid ratio was also associated to larger annual increases in the cognitive-functional scale CDR-SOB, of 0.46 (0.22–0.71) in Aβ1–42[−]Ratio[+] and 0.65 (0.46-0.85) in Aβ1–42[+]Ratio[+], compared to 0.27 (0.16–0.39) in Aβ1–42[−]Ratio[−] and no significant changes in Aβ1–42[+]Ratio[−].

## Discussion

The results of our study indicate that, compared to Aβ1–42 alone, the Aβ1–42/Aβ1–40 CSF ratio is more strongly associated with tau markers and with cognitive and functional progression. Regardless of the Aβ1–42 status, participants with low CSF Aβ1–42/Aβ1–40 ratio showed a biochemical and clinical profile characterized by increased levels of CSF tTau and pTau181, worse functional prognosis and larger cognitive decline. Interestingly, when the Aβ1–42/Aβ1–40 ratio was normal, participants with low Aβ1–42 levels had a biochemical and clinical profile similar to that of participants with normal Aβ1–42. Our findings provide evidence suggesting that the use of the Aβ1–42/Aβ1–40 ratio is less confounded by other comorbidities or processes, and thus, this work favors the use of the Aβ1–42/Aβ1–40 ratio as a marker of AD in clinical laboratories and in clinical trials.

The use of CSF Aβ1–42 alone as a marker of amyloid plaques entails some limitations. Numerous studies have reported that low concentrations of Aβ1–42 can be found in some non-AD conditions such as prionopathies, bacterial meningitis, inflammatory diseases, amyloid angiopathy, or frontotemporal dementia [10–14, 39], thus limiting its diagnostic accuracy. Different hypotheses have been suggested to explain these findings. Among other possibilities, low levels of Aβ1–42 in these contexts could be in relation with a decrease in Aβ generation due to neuronal or synaptic loss [10, 15] or the consequence of abnormal clearance through the blood-brain barrier [10]. As Aβ1–40 would be similarly affected by these processes, the use of the Aβ1-42/Aβ1-40 ratio could compensate the reduction in these situations to some extent, thus being less influenced by these processes and reflecting more accurately the presence of amyloid plaques. The stronger association of the Aβ1–42/Aβ1–40 ratio to other AD markers (tau markers) is in line with these hypotheses. Another limitation for the use of Aβ1–42 alone is that it is particularly sensitive to preanalytical and analytical variations, such as changes in the material of collection or storage tubes, number of freeze-thaw cycles, and volume of aliquoted CSF for storage [33, 40, 41]. In our study, we took advantage of a large cohort of subjects with various neurodegenerative and non-neurodegenerative conditions where CSF was collected using the same preanalytical protocol and analyzed under the same standard operating procedures. We found that the overall agreement between Aβ1–42 and the Aβ1–42/Aβ1–40 ratio did not exceed 85% in the whole sample, regardless of the cutoffs definition, suggesting that both markers might be tracking similar but not identical processes or that they are influenced differently by other factors.

We found that CSF concentrations of tTau and pTau181 were higher in the presence of low Aβ1–42/Aβ1–40 ratio, regardless of the Aβ1–42 status. This association was present in the whole sample but also within each diagnostic group. Thus, in the groups of CN, AD, and Down syndrome, less likely affected by non-AD pathology, low Aβ1–42/Aβ1–40 ratio values, but not low Aβ1–42 levels alone, were associated with high concentrations of markers of neurofibrillary pathology and neurodegeneration (pTau181, tTau, and NfL). In other contexts (DLB, FTLD, and other diagnoses), low levels of Aβ1–42 were only associated with markers of neurofibrillary pathology in the presence of reduced Aβ1–42/Aβ1–40 ratio. Our findings indicate that the ratio is more strongly associated to the AD pathophysiological process (both as main and comorbid pathology) and also support the idea that the isolated reduction of Aβ1–42 levels in CSF might reflect additional processes (such as neuronal or synaptic loss) or that the chrono pathology of their changes along the disease is not identical. These results are in line with our previous study showing that the Aβ1–42/Aβ1–40 ratio presents a stronger correlation with cerebral amyloid burden than Aβ1–42 alone [22].

Baseline levels of the Aβ1–42/Aβ1–40 ratio were also influential in the cognitive and functional outcomes of participants in our study. We found that participants with low Aβ1–42/Aβ1–40 ratio had faster cognitive and functional worsening, especially in the group with low Aβ1–42 but also when Aβ1–42 was in the normal range. The group with a low Aβ1–42/Aβ1–40 ratio also presented a more rapid decline in episodic memory measured by the free and cued selective reminding test. These findings support the use of the Aβ1–42/Aβ1–40 ratio over Aβ1–42 alone in the prognostic assessment of patients with cognitive decline.

The major strengths of our study are the large sample size and the inclusion of a variety of diagnoses. Another relevant strength is the fact that the same standard operating procedures were used in the processing and analysis of all samples. Aβ1–42 and Aβ1–40 were measured simultaneously, and we followed the same preanalytical and analytical protocol in all samples, thus minimizing the impact of confounders, which are critical in the case of amyloid-β peptides. Lastly, we replicated our results by using different levels of cutoffs, defined as percentiles from a cognitively normal population. This approach allowed us to match pairs of cutoffs for Aβ1–42 and Aβ1–42/Aβ1–40 ratio that had comparable levels of sensitivity and specificity. But we also acknowledge some limitations.

## Limitations of the study

Despite the large sample size, extensive cognitive repeated measures were not available for all participants, thus limiting the statistical power in the longitudinal analysis. On the other hand, amyloid PET imaging was only available in a reduced group of participants previously reported [22].

## Conclusions of the study

The present work highlights the importance of routinely measuring Aβ1–40 in the CSF in combination with Aβ1–42 to assess the Aβ1–42/Aβ1–40 ratio, as this measure reflects more accurately the presence of amyloid plaques and is a useful and robust tool for the diagnostic and prognostic evaluation of patients with cognitive decline. The fact that the Aβ1–42/Aβ1–40 ratio shows a stronger association than Aβ1–42 with markers of neurofibrillary pathology and with cognitive and functional decline strengths the utility of this ratio in the clinical context of symptomatic and preclinical AD but also to detect concomitant pathology in other neurodegenerative diseases.

## Supplementary Information

**Additional file 1.** Supplementary tables and figures.

## Data Availability

Raw anonymized data and code for the statistical analysis are available upon reasonable request. All requests should be sent to the corresponding author detailing the study hypothesis and statistical analysis plan. The steering committee of this study will decide whether data/code sharing is appropriate based on the novelty and scientific rigor of the proposal. All applicants will be asked to sign a data access agreement.
