# Supplementary material for: The Aβ1–42/Aβ1–40 ratio in CSF is more strongly associated to tau markers and clinical progression than Aβ1–42 alone
Source: Alzheimers Res Ther. 2022 Feb 1;14:20. doi: 10.1186/s13195-022-00967-z (PMC8809019; doi:10.1186/s13195-022-00967-z)

SUPPLEMENTARY MATERIAL

Table of Contents

Supplementary Table 1. Characteristics of the reference population of cognitively normal participants 2

Supplementary Table 2. Quantile-based cutoffs used in this study 3

Supplementary Table 3. Characteristics of the subset of participants included in the prognostic assessment 4

Supplementary Figure 1. CSF biomarkers across clinical groups 5

Supplementary Figure 2. Distribution of participants according to their CSF levels of Aβ1–42 and Aβ1–42/Aβ1–40 ratio within stages 6

Supplementary Figure 3. Distribution of participants according to their CSF levels of Aβ1–42 and Aβ1–42/Aβ1–40 ratio with different cutoff definitions 7

Supplementary Figure 4. Agreement of Aβ1–42 and the Aβ1–42/Aβ1–40 ratio with tTau, pTau and NfL 8

Supplementary Figure 5. Agreement of Aβ1–42 and the Aβ1–42/Aβ1–40 ratio with tTau, pTau and NfL in the groups of CN, AD and Down participants 9

Supplementary Figure 6. Correlation of Aβ1-42 and Aβ1-42/Aβ1-40 ratio with other CSF biomarkers 10

Supplementary Figure 7. Correlation of Aβ1-42 and Aβ1-42/Aβ1-40 ratio with other CSF biomarkers within clinical stages 11

Supplementary Figure 8. Correlation of Aβ1-42 and Aβ1-42/Aβ1-40 ratio with other CSF biomarkers within amyloid profiles 12

Supplementary Figure 9. Levels of tTau (A), pTau181 (B) and NfL (C) in CSF according to their amyloid profile stratified by diagnosis 13

Supplementary Figure 10. Levels of tTau (A), pTau181 (B) and NfL (C) in CSF according to their amyloid profile stratified by clinical stage 14

Supplementary Figure 11. Levels of tTau, pTau181 and NfL in CSF according to their amyloid profile when different cutoffs were applied 15

Supplementary Figure 12: Estimation of the annual change in cognitive and functional scores across amyloid profiles stratified by diagnosis 16

Supplementary Table 1. Characteristics of the reference population of cognitively normal participants

CSF cutoff points for Aβ1–42 and the Aβ1–42/Aβ1–40 ratio for this study were defined by calculating a range of quantiles for each biomarker in a cognitively normal middle-aged population (below 60 years old) of 69 volunteers of the SPIN cohort. All volunteers in the SPIN cohort received a standard neuropsychological evaluation that was in normal range to ensure that cognition was preserved. Cerebrospinal fluid was obtained from all volunteers following the same standard preanalytical protocol followed in the present study. CSF levels of core AD biomarkers (Aβ1–42, Aβ1–40, tTau, and pTau181) were measured in the Lumipulse fully-automated platform (Fujirebio Europe, Ghent, Belgium).

More details on the SPIN cohort protocol and inclusion/exclusion criteria can be found in Alcolea D et al. Alzheimers Dement (N Y) 2019.

N=69	Mean (SD)	Range
**Age, years**	48.6 (9.67)	23-60
**Female / Male (% Female)**	46/23 (66.7%)	-
**MMSE score**	29.3 (0.905)	27-30
**Education, years**	17 (3.58)	8-20
**Follow-up time, years**	2.67 (2.22)	0-7.08
**APOE4+ / APOE4- (%APOE4+)**	24/45 (34.8%)	-
**CSF Aβ1-42 , pg/ml**	1154 (355)	597-2034
**CSF Aβ1-40 , pg/ml**	11368 (2889)	5657-17404
**CSF Aβ1-42 / Aβ1-40**	0.101 (0.0143)	0.045-0.121
**CSF tTau, pg/ml**	232 (72.7)	111-404
**CSF pTau181, pg/ml**	33.2 (10.5)	18.2-66.8
**CSF NfL, pg/ml**	350 (136)	104-725

Supplementary Table 2. Quantile-based cutoffs used in this study

As described in the Methods section, different cutoff levels were tested in this study. The framed row indicates the level that was used to present the main results and correspond to 95% quantile values in a cognitively normal population of participants < 60 years. Percentile scales for Aβ1–42 and the Aβ1–42/Aβ1–40 ratio are reversed.

Reference population: Cognitively normal participants <60 years

Quantile	Aβ1-42 (pg/ml)	Aβ-42 / Aβ1-40 ratio	tTau (pg/ml)	pTau181 (pg/ml)	NfL (pg/ml)
**2.5%**	1898	0.119	116	18.6	147
**5%**	1828	0.117	131	19.4	165
**10%**	1663	0.114	141	21.3	189
**20%**	1396	0.111	166	23.7	220
**30%**	1317	0.108	189	26	287
**50%**	1107	0.104	218	31.8	347
**70%**	929	0.102	273	38.3	371
**80%**	829	0.098	293	42.6	466
**90%**	732	0.082	327	46.4	538
**95%**	**637**	**0.07**	**357**	**52.2**	**554**
**97.5%**	617	0.06	376	54.6	582

Reference population: Cognitively normal participants >50 years

Quantile	Aβ1-42 (pg/ml)	Aβ1-42 / Aβ1-40 ratio	tTau (pg/ml)	pTau181 (pg/ml)	NfL (pg/ml)
**2.5%**	1960	0.121	141	20.5	218
**5%**	1813	0.12	145	21.9	267
**10%**	1678	0.115	174	24.5	287
**20%**	1569	0.11	191	27.1	350
**30%**	1396	0.108	220	32.5	375
**50%**	1166	0.103	268	39.8	508
**70%**	880	0.094	306	46.6	561
**80%**	779	0.072	333	53.3	606
**90%**	667	0.058	418	63.2	721
**95%**	604	0.05	502	86.8	1193
**97.5%**	544	0.045	659	109.9	1232

Supplementary Table 3. Characteristics of the subset of participants included in the prognostic assessment

CN: cognitively normal; AD: Alzheimer's disease; DLB: dementia with Lewy bodies; FTLD: frontotemporal lobar degeneration-related syndrome; MMSE : Mini-mental state examination.

	AD	DLB	FTLD	Other
**N**	296	60	90	348
**AGE, years**	74 [70-77.2]	75 [70.8-79]	70 [65-74]	71 [65-76]
**SEX, females/males (%females)**	181/115(61.1%)	35/25(58.3%)	35/55(38.9%)	199/148(57.3%)
**MMSE score**	26 [24-27]	27 [24-28]	27 [25-28]	27 [25-28]
**Education, years**	10 [8-13]	8 [6.75-12]	12 [9-17.8]	10 [8-14]
**APOEε4, APOEε4-/ APOEε4+ (%APOEε4+)**	149/141(51,4%)	14/46(23,3%)	19/68(21,8%)	68/277(19,7%)
**Follow-up, years**	0.567 [0-2.28]	3.68 [2.52-4.42]	1.68 [1.04-2.96]	0 [0-1.15]
**Aβ1–42, pg/ml**	568 [448-684]	728 [552-1110]	892 [626-1229]	1002 [689-1367]
**Aβ1–40, pg/ml**	13152 [10898-15467]	10990 [8897-13936]	10320 [8174-14531]	11376 [8618-14313]
**Aβ1–42 / Aβ1–40**	0.0439 [0.0368-0.05]	0.0735 [0.0514-0.102]	0.0979 [0.0765-0.104]	0.0961 [0.0692-0.105]
**tTau, pg/ml**	638 [485-863]	340 [248-490]	314 [218-433]	290 [215-370]
**pTau181, pg/ml**	102 [78.4-140]	47.7 [34.5-78.8]	39.2 [28.8-53.2]	41.2 [30.4-52.1]
**NfL, pg/ml**	931 [732-1114]	736 [640-905]	1186 [640-2141]	1295 [690-1995]
**Amyloid profile (Aβ1-42[-]Ratio[-] / Aβ1-42[-]Ratio[+] / Aβ1-42[+]Ratio[-] / Aβ1-42[+]Ratio[+])**	2/96/1/197	30/8/2/20	62/5/9/14	242/32/16/58

Supplementary Figure 1. CSF biomarkers across clinical groups

CN: cognitively normal; AD: Alzheimer's disease; DLB: dementia with Lewy bodies; FTLD: frontotemporal lobar degeneration-related syndrome. Horizontal dashed lines indicate 95% quantile values (Q95%) for Aβ1–42 and Aβ1–42/Aβ1–40 in a middle-aged cognitively normal population as described in the Methods section.

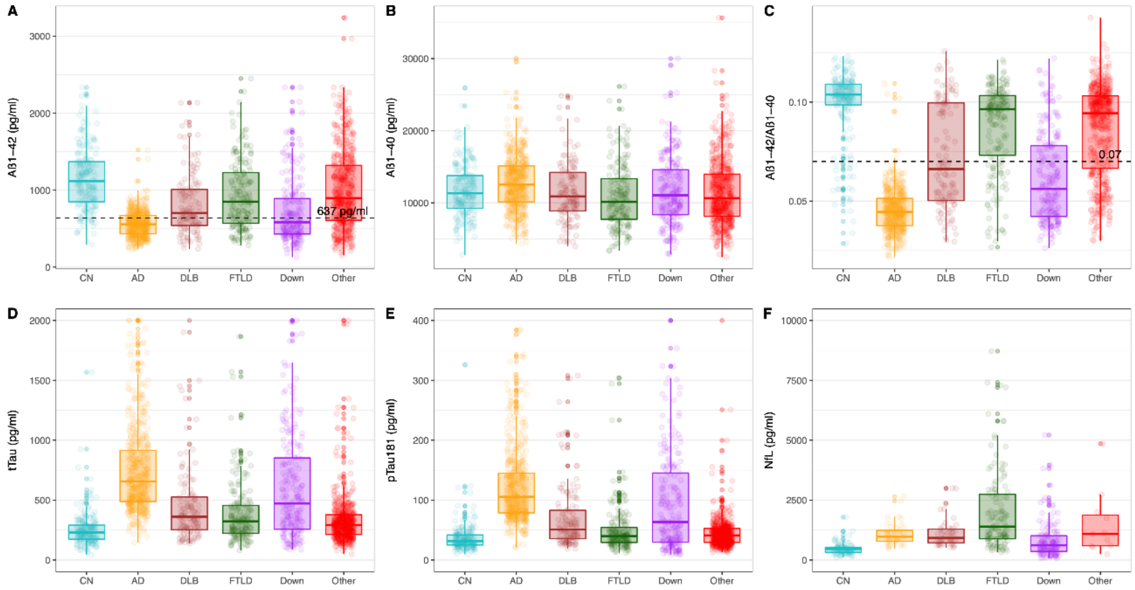

Supplementary Figure 2. Distribution of participants according to their CSF levels of Aβ1–42 and Aβ1–42/Aβ1–40 ratio within stages

CN: cognitively normal; AD: Alzheimer's disease; DLB: dementia with Lewy bodies; FTLD: frontotemporal lobar degeneration-related syndrome; MCI: mild cognitive impairment. Dashed lines indicate 95% quantile values (Q95%) for Aβ1–42 and Aβ1–42/Aβ1–40 in a middle-aged cognitively normal population as described in the Methods section.

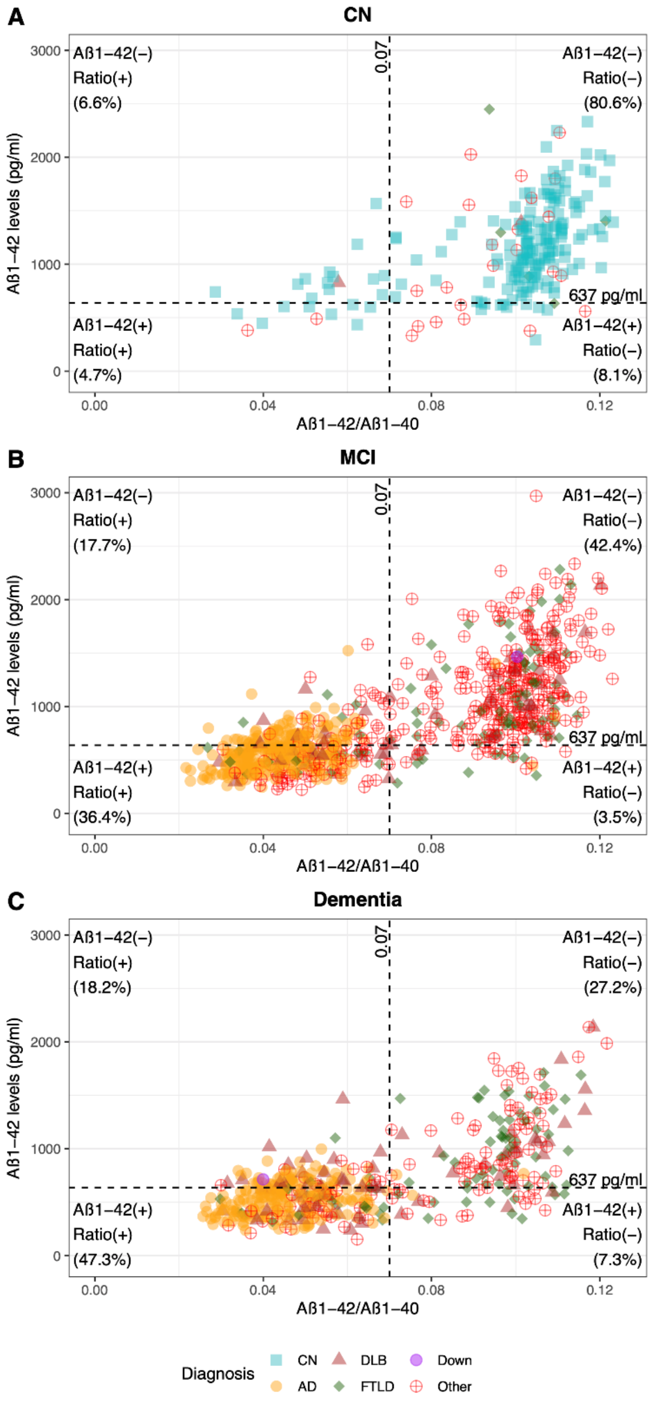

Supplementary Figure 3. Distribution of participants according to their CSF levels of Aβ1–42 and Aβ1–42/Aβ1–40 ratio with different cutoff definitions

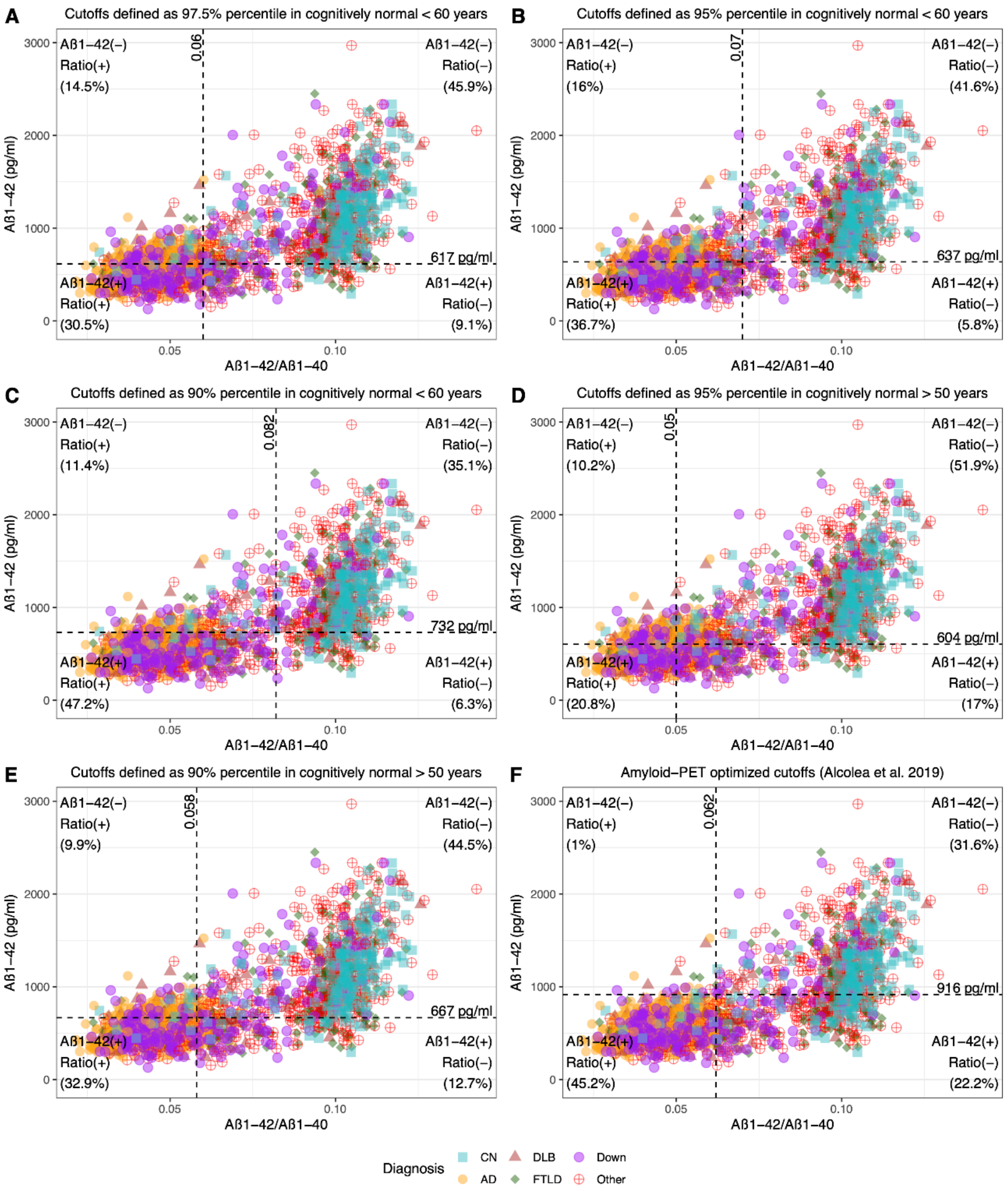

Supplementary Figure 4. Agreement of Aβ1–42 and the Aβ1–42/Aβ1–40 ratio with tTau, pTau and NfL

CN: cognitively normal; AD: Alzheimer's disease; DLB: dementia with Lewy bodies; FTLD: frontotemporal lobar degeneration-related syndrome; MCI: mild cognitive impairment. Dashed lines indicate 95% quantile values (Q95%) in a middle-aged cognitively normal population as described in the Methods section.

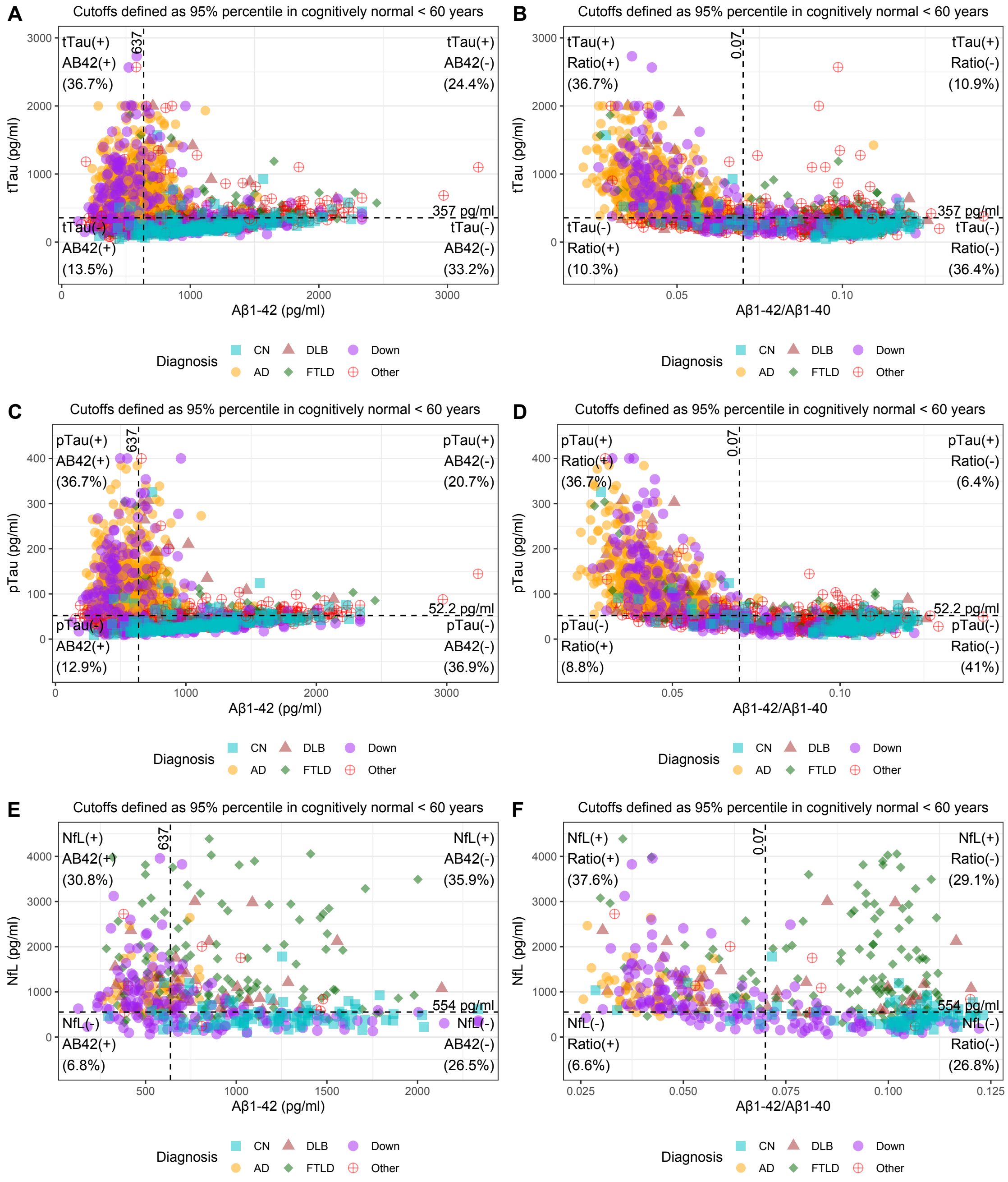

Supplementary Figure 5. Agreement of Aβ1–42 and the Aβ1–42/Aβ1–40 ratio with tTau, pTau and NfL in the groups of CN, AD and Down participants

CN: cognitively normal; AD: Alzheimer's disease.Dashed lines indicate 95% quantile values (Q95%) in a middle-aged cognitively normal population as described in the Methods section.

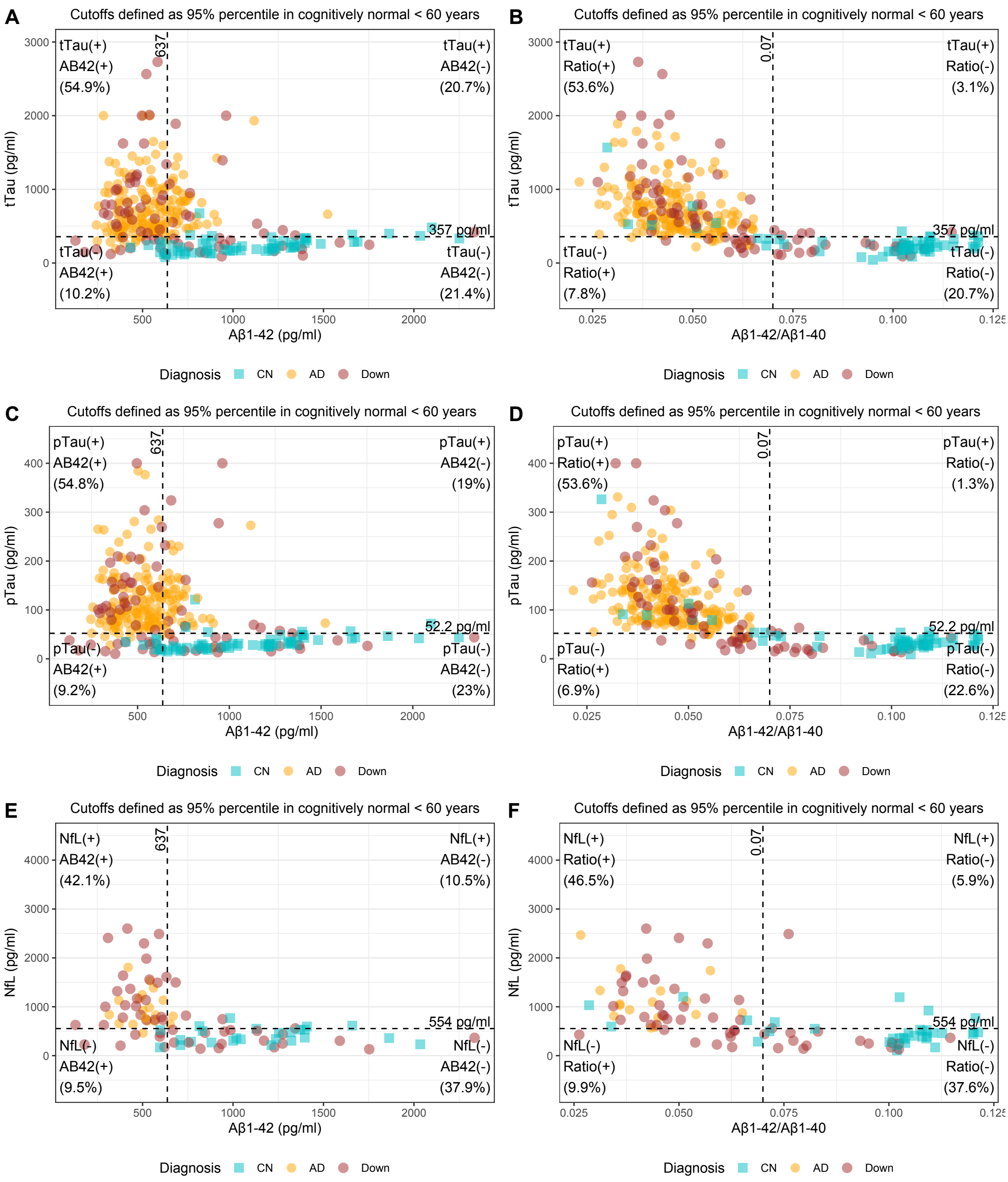

Supplementary Figure 6. Correlation of Aβ1-42 and Aβ1-42/Aβ1-40 ratio with other CSF biomarkers

Values in the cells correspond to Spearman's Rho coefficient. Shaded cells indicate significant correlations after adjustment for multiple comparisons. All paired correlations were assessed, but only significant correlations are displayed. CN: Cognitively normal; AD: Alzheimer's disease; DLB: dementia with Lewy bodies; FTLD: Frontotemporal lobar degeneration-related syndromes.

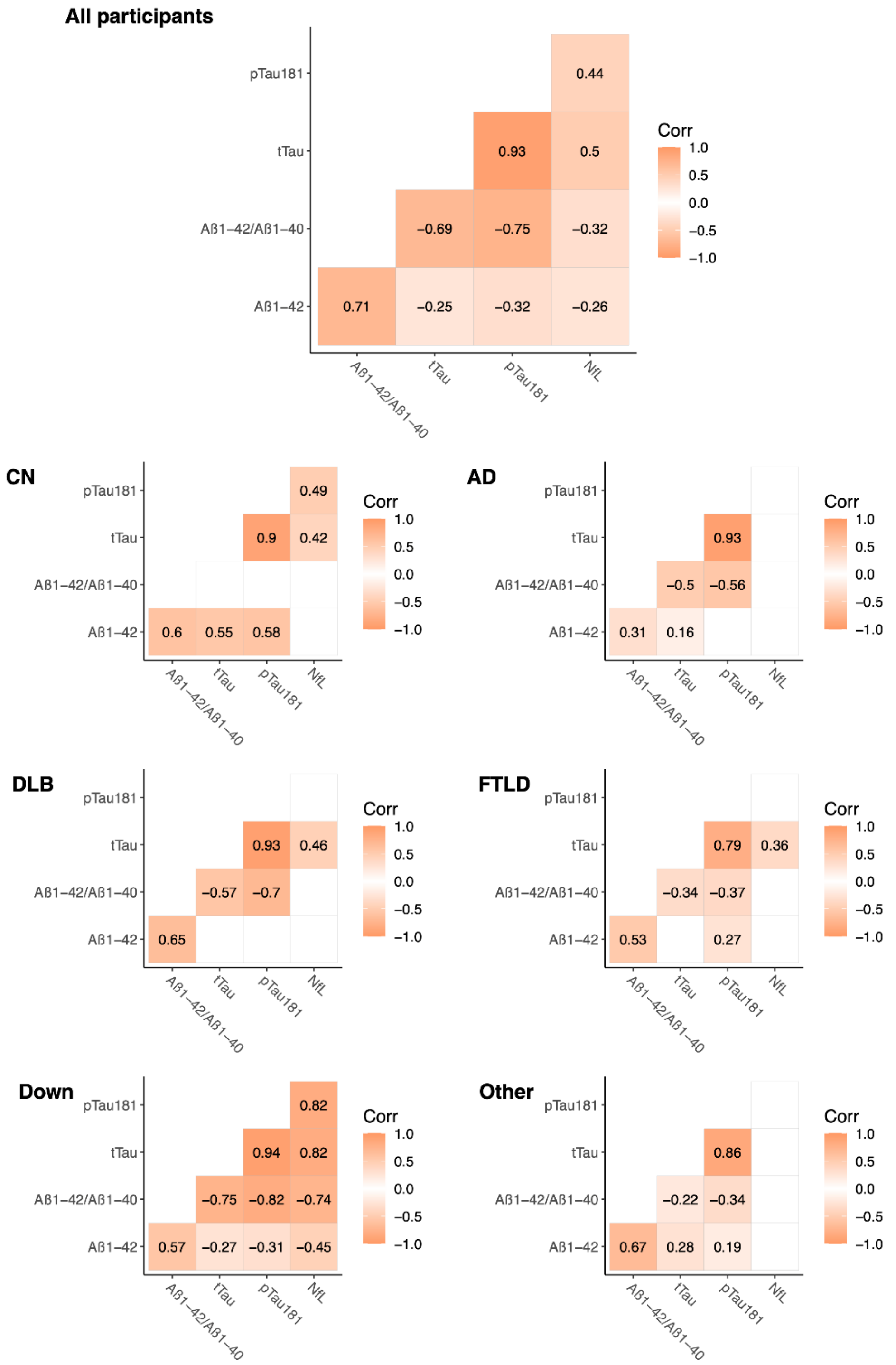

Supplementary Figure 7. Correlation of Aβ1-42 and Aβ1-42/Aβ1-40 ratio with other CSF biomarkers within clinical stages

Values in the cells correspond to Spearman's Rho coefficient. Shaded cells indicate significant correlations after adjustment for multiple comparisons. All paired correlations were assessed, but only significant correlations are displayed. CN: Cognitively normal; MCI: mild cognitive impairment.

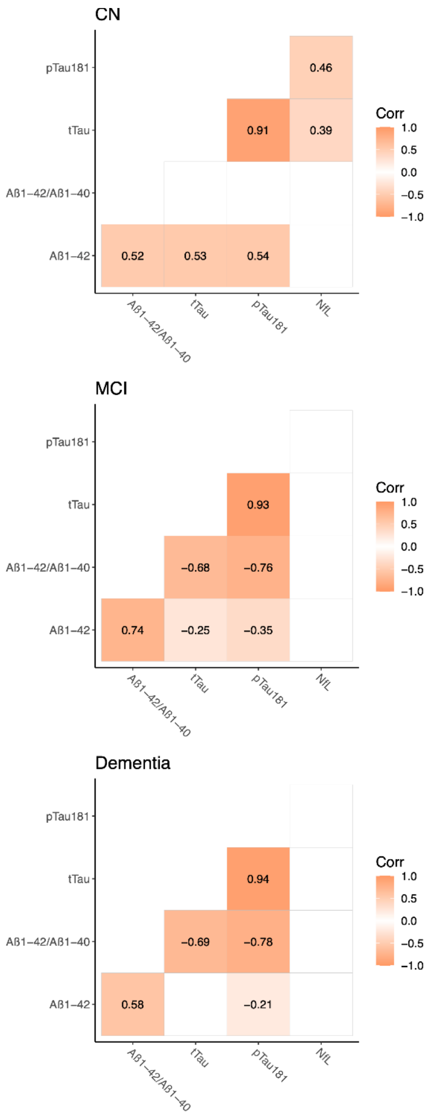

Supplementary Figure 8. Correlation of Aβ1-42 and Aβ1-42/Aβ1-40 ratio with other CSF biomarkers within amyloid profiles

Values in the cells correspond to Spearman's Rho coefficient. Shaded cells indicate significant correlations after adjustment for multiple comparisons. All paired correlations were assessed, but only significant correlations are displayed.

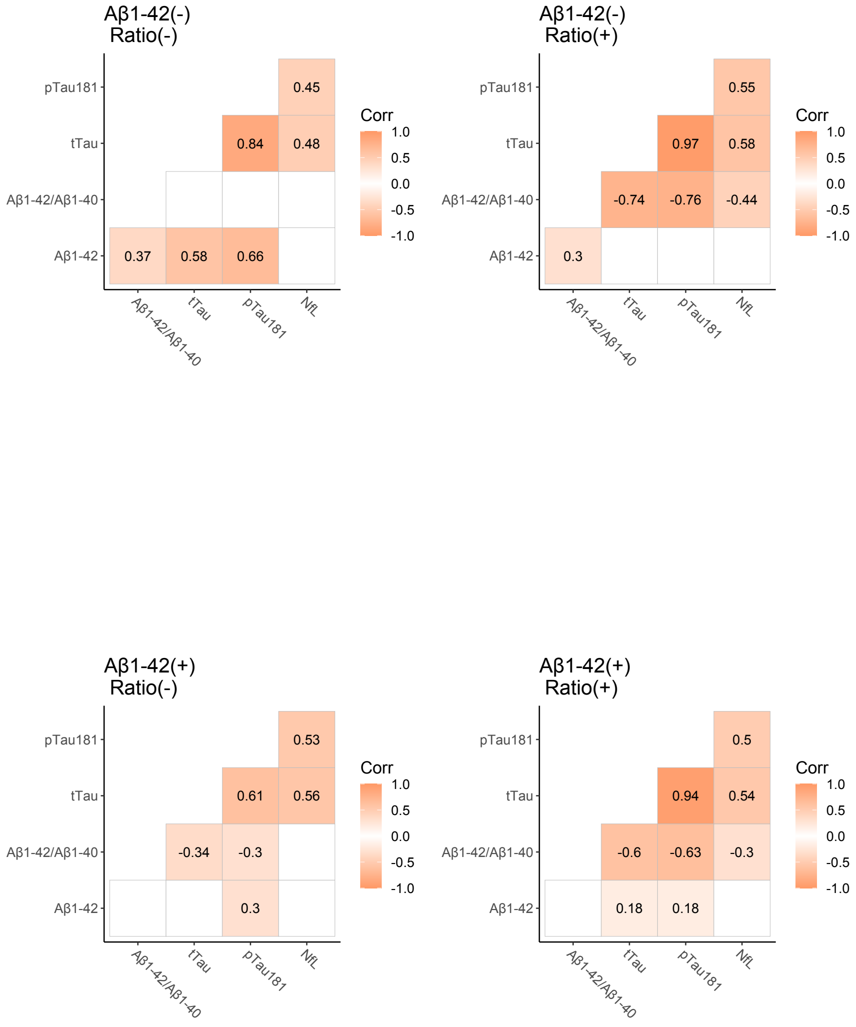

Supplementary Figure 9. Levels of tTau (A), pTau181 (B) and NfL (C) in CSF according to their amyloid profile stratified by diagnosis

CN: Cognitively normal; AD: Alzheimer's disease; DLB: dementia with Lewy bodies; FTLD: Frontotemporal lobar degeneration-related syndromes.

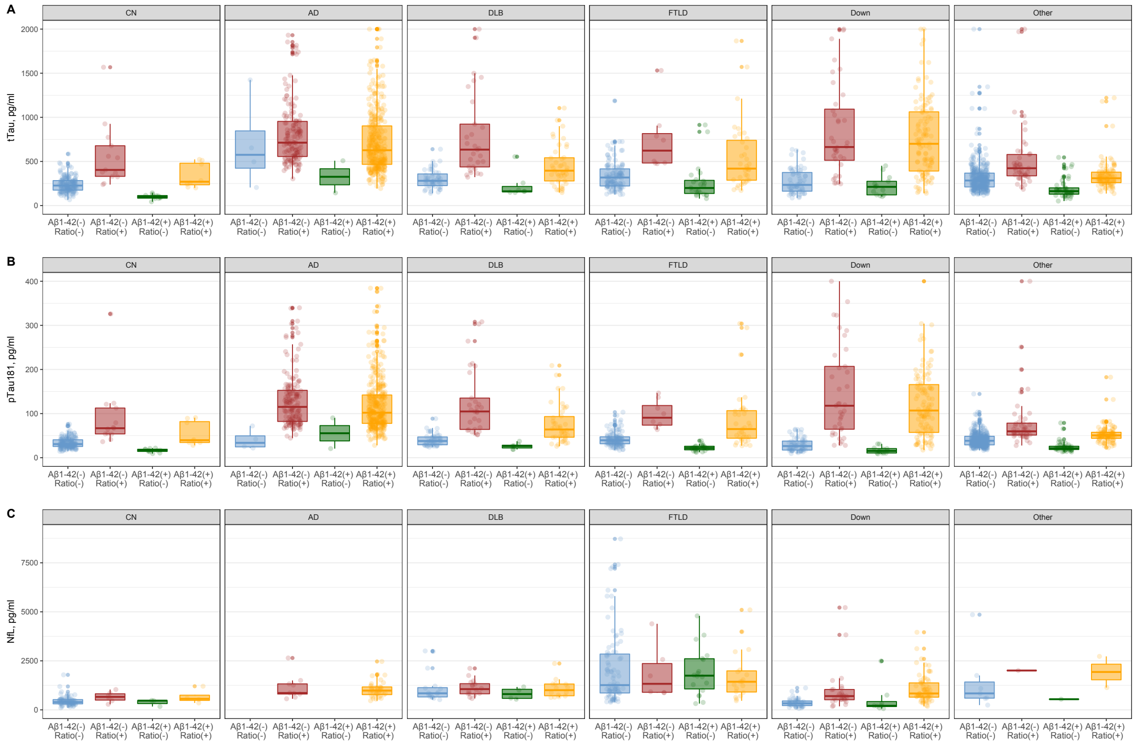

Supplementary Figure 10. Levels of tTau (A), pTau181 (B) and NfL (C) in CSF according to their amyloid profile stratified by clinical stage

CN: Cognitively normal; MCI: Mild cognitive impairment.

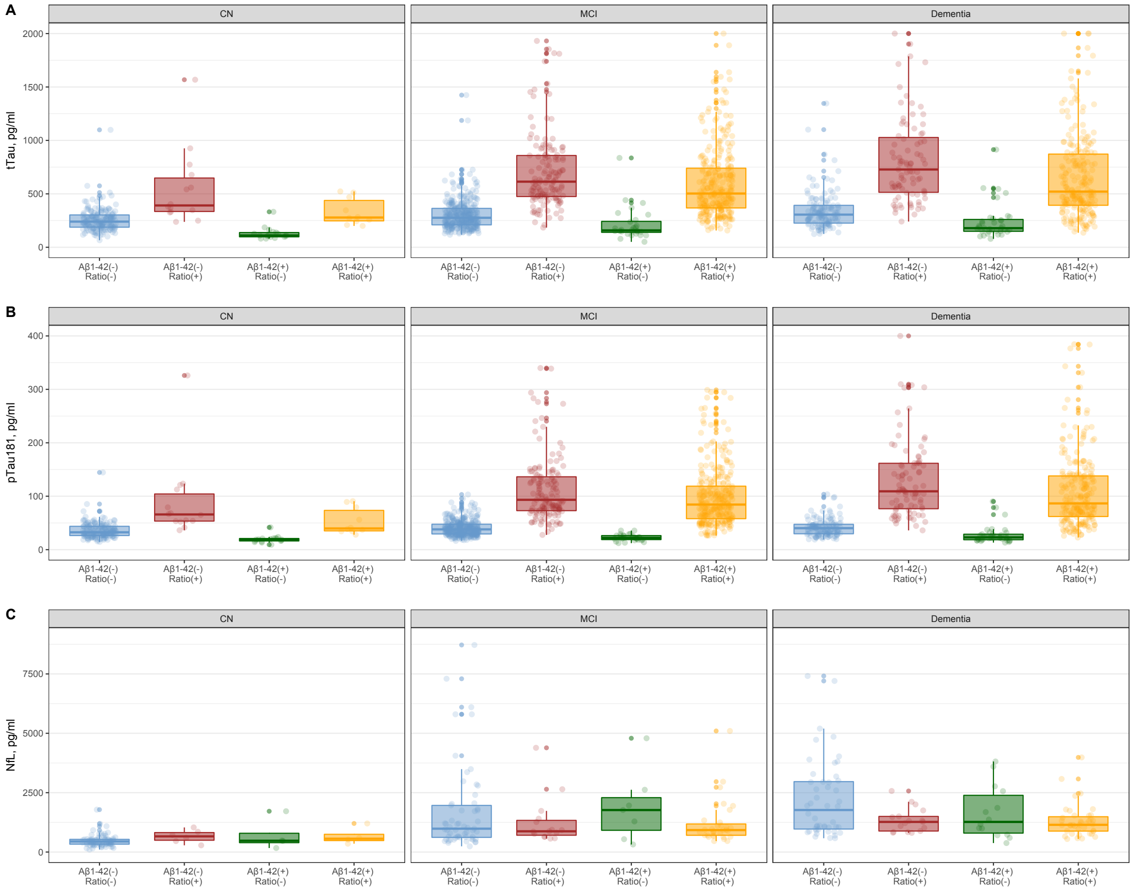

Supplementary Figure 11. Levels of tTau, pTau181 and NfL in CSF according to their amyloid profile when different cutoffs were applied

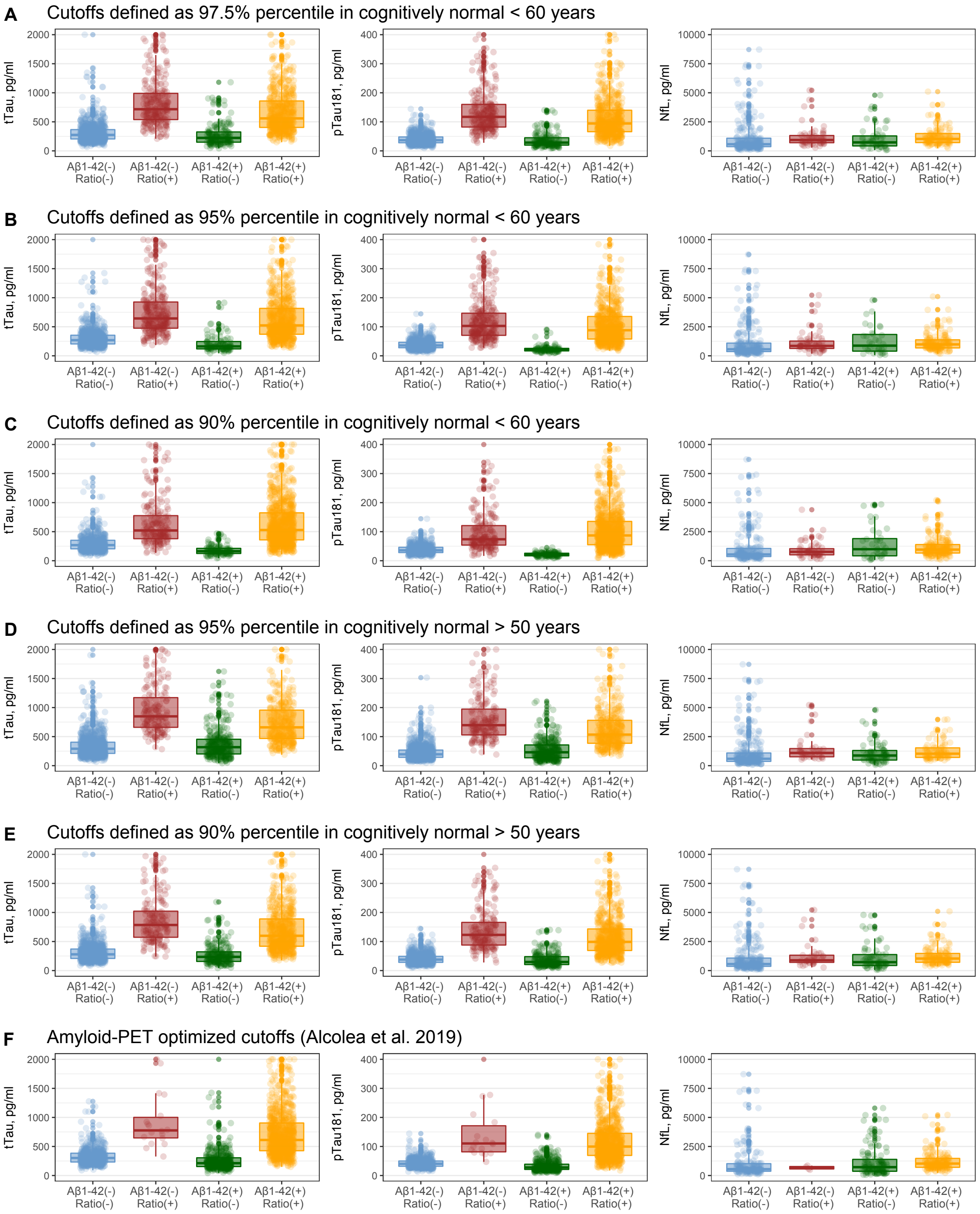

Supplementary Figure 12: Estimation of the annual change in cognitive and functional scores across amyloid profiles stratified by diagnosis

Estimations of the annual change in MMSE (A), CDR-SOB (B) and FCSRT total score (C) were calculated through linear-mixed models adjusted by baseline MMSE score, baseline age, sex, years of education, pTau181 levels, *APOEε4* status and diagnosis. MMSE: Mini-Mental State Examination; CDR-SOB: Clinical Dementia Rating Sum of Boxes; FCSRT: Free and cued selective reminding test; CN: Cognitively normal; AD: Alzheimer's disease; DLB: dementia with Lewy bodies; FTLD: Frontotemporal lobar degeneration-related syndromes.

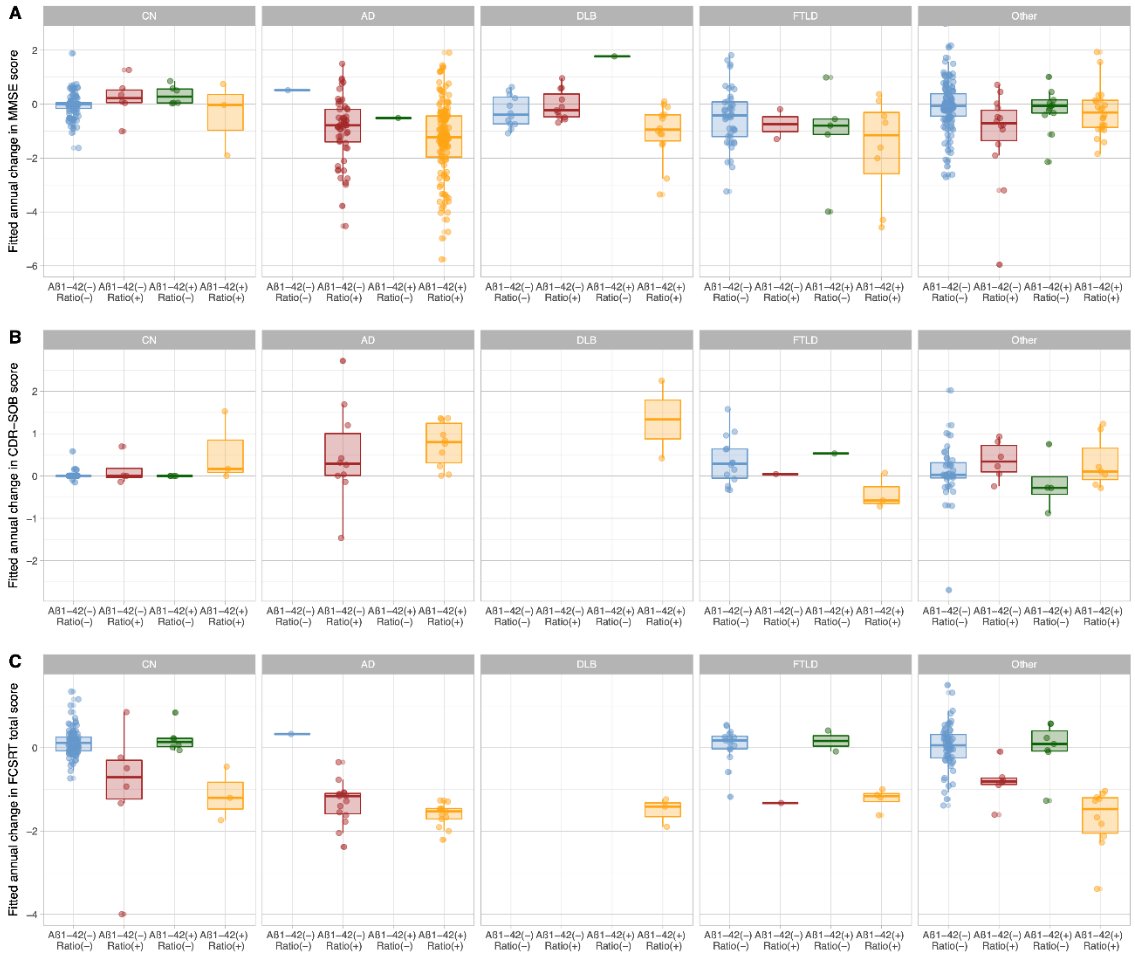

Supplement: Supplementary file 1 — Additional file 1. Supplementary tables and figures. [file 13195_2022_967_MOESM1_ESM.pdf]
